# Low-Resolution Neurocognitive Aging and Cognition: An Embodied Perspective

**DOI:** 10.3389/fnsys.2021.687393

**Published:** 2021-07-27

**Authors:** Jordan Mille, Simona M. Brambati, Marie Izaute, Guillaume T. Vallet

**Affiliations:** ^1^CNRS, LAPSCO (UMR CNRS 6024), Université Clermont Auvergne, Clermont-Ferrand, France; ^2^CRIUGM, Université de Montréal, Montréal, QC, Canada

**Keywords:** embodiment, aging, neurocognition, perceptual processing, conjunction, distinctiveness

## Abstract

Consistent with embodied cognition, a growing evidence in young adults show that sensorimotor processing is at the core of cognition. Considering that this approach predicts direct interaction between sensorimotor processing and cognition, embodied cognition may thus be particularly relevant to study aging, since this population is characterized by concomitant changes in sensorimotor and cognitive processing. The present perspective aims at showing the value and interest to explore normal aging throughout embodiment by focusing on the neurophysiological and cognitive changes occurring in aging. To this end, we report some of the neurophysiological substrates underpinning the perceptual and memory interactions in older adults, from the low and high perceptual processing to the conjunction in the medial temporal lobe. We then explore how these changes could explain more broadly the cognitive changes associated with aging in terms of losses and gains.

## Introduction

Embodied cognition defines the body and the interaction with the world as shaping cognition and not just as simple inputs/outputs (Wilson, 2002). As a consequence, perceptual and motor systems should play a crucial role in cognitive functioning. Growing evidence, especially in young adults, has shown that sensorimotor components are at the core of language (Pulvermüller et al., 2005), attention (Bradley, 2007), memory (Versace et al., 2014) or action (Hommel, 2009). However, few studies are conducted in normal aging (Vallet, 2015). This is particularly surprising given that aging is marked by perceptual (and motor) decline in the one hand and by cognitive decline in the other hand. This perspective aims at proposing an embodied account of age-related cognitive decline focusing on perceptual and memory interactions in older adults. To this end, the neurophysiological substrates at the origin of the interactions between perceptual and memory should be understood. The low and high levels of sensory neurophysiological changes in older adults will be firstly described as their impact on the emergence of memory representations. Then, with the support of the hierarchical representational model (Murray and Bussey, 1999; Saksida and Bussey, 2010), we will examine how changes in the perceptual-mnemonic conjunctive processes, occurring in the medial temporal lobe (MTL), alter the emergence of representation. Finally, we will discuss how these changes could explain more broadly the cognitive changes associated with aging in terms of losses and gains based on the Activation-Integration model (Versace et al., 2009, 2014).

## Sensory-Perceptual Decline and Low-Resolution Representation in Aging

Biological aging affects the whole body including, at a low-sensory level (sensory organs), many sensory modalities. Recently, the five Aristotelian senses (hearing, vision, taste, touch, and smell) was simultaneously assessed in older adults aged 57–85 years (Correia et al., 2016). The results showed that 74% of the participants had a deficit in identifying taste, 70% in touch, 22% in smell, 20% in corrected vision and 18% in corrected hearing. This study also provides for the first time an estimate of the proportion in which the sensory modalities are jointly altered. Two thirds of the participants had a deficit of two or more modalities, 27% had a deficit of only one of these modalities, while only 6% showed no impairment. At a higher level, aging worsens the transmission of sensory information from these organs to the brain (Ulfhake et al., 2002) and higher perceptual thresholds are also found (e.g., Fozard and Gordon-Salant, 2001). At a cortical level, the occipital sensory cortex is less affected in aging with regard to structural integrity (e.g., Peters, 2006), but long-term peripheral sensory alteration may promote atrophy of the perceptual areas of the brain (Baltes and Lindenberger, 1997; Boucard et al., 2009; Golub, 2017). Functionally, the dopaminergic modulation deficit in aging, regulating the neurons sensitivity to related signals, reduces the functional specialization of neuronal activation. This is also true in neuronal circuits that are still relatively intact (Li et al., 2001), especially for visual stimuli in posterior regions (Park et al., 2004). The impoverished perceptual signal results in weakened unisensory and strengthen multisensory information processing (de Dieuleveult et al., 2017). As older adults exhibited reduced activity in occipital regions coupled with increased frontal activity, a functional compensation occurs (Davis et al., 2008).

**FIGURE 1:**
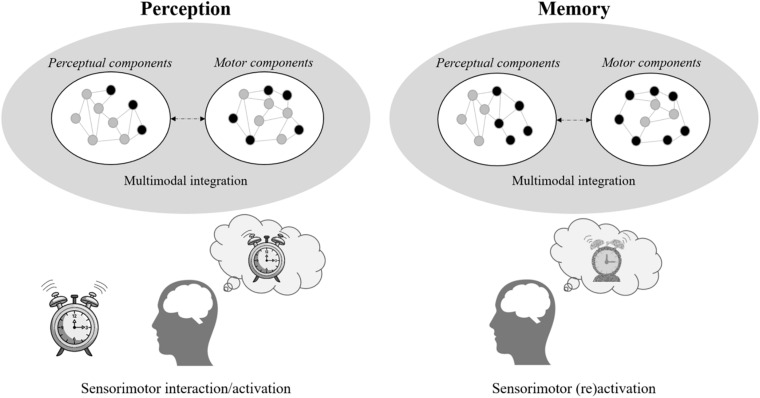
Illustration of the sensorimotor grounded memory traces. The left panel represents the activation of the sensorimotor components when seeing an object (here, an alarm clock). The right panel represents the partial (re)activation, constrained by the present situation, of these sensorimotor components leading to the emergence of the associated memory. As memory is defined as a dynamic emergence, the representation may be slightly different from the percept.

Embodied cognition predicts that these perceptual changes should directly impact cognitive functioning. In this approach, all forms of knowledge (e.g., semantic, episodic) remains grounded in its sensorimotor components (Glenberg et al., 2013). The cognitive representations are not retrieved in memory, but instead emerge from the simulation of these components associated with the individual’s previous experiences based on the constraints of the present situation (Figure 1). Simulation refers here to the automatic and mandatory re-enactment of the brain activities of the perceptual, motor and emotional states produced by past experiences in the modal and heteromodal areas (Barsalou, 2008). Thus, the neurophysiological degradation occurring in aging in perceptual processing should deteriorate the simulation mechanism at the core of representation emergence (Vallet, 2015).

A less efficient simulation in older adults should lead to the emergence of “low-resolution” impoverished representations, that is, representations with lower details. In other words, the signal-to-noise ratio of the sensorimotor simulation should be lower, mainly due to a decrease in central perceptual processing. Whereas the perceptual discriminability in memory is underlied by occipital regions regardless of age (Bowman et al., 2019), older adults exhibits reduced representation fidelity in these regions (Zheng et al., 2018). Impoverished sensory input may decrease the activation of specific sensory components of perceptually present information, but it should generally not alter the ability to simulate perceptually absent (and therefore mnemonic) information. Coherently, it has been shown that older adults did not suffer from a retrieval deficit, but insteaded exhibit less precise mnemonic representations of the items (i.e., less accurate responses on the color and orientation) and of the context (e.g., location) of the information to be learned (Korkki et al., 2020). This study also showed that the reduction in accuracy is not fully explained by a deficit in low-level sensory functioning alone (visual acuity), which may rather occur from neural dedifferentiation in the parahippocampus (Koen et al., 2019). The lower-resolution hypothesis could also account for associative (e.g., source memory) deficits in aging. Indeed, older adults have the greatest deficit in access associations requiring a high level of specificity (e.g., the old man was in this park), whereas they performed as well as younger adults to recognize general associations (e.g., the old man was in a park) and fuzzy associations (e.g., the old man was out somewhere) (Greene and Naveh-Benjamin, 2020). As all forms of knowledge (e.g., semantic, episodic, autobiographical) emerges from simulation, a consequence of this hypothesis is that memory deficits in older people should not be limited to newly learned knowledge. Coherently, older adults also recall less specific perceptual or spatiotemporal details in autobiographical memory tasks (very long-term memory see, Frankenberg et al., 2021). A better understanding of the changes requires to study the neurophysiological mechanisms underlying memory in the MTL (the hippocampus and surrounding cortex) and the effect of age on them.

## Low-Resolution Representations Induce More Interferences in Aging Memory

In the MTL, the general coding principle is based on the functional theory of the hippocampus (e.g., Marr, 1971; Rolls, 2013). According to this theory, the emergence of specific memories (i.e., episodic memory) relies on the pattern separation (PS) and the pattern completion (PC) mechanisms. PS is defined as the ability to reduce interference from similar percepts by processing non-overlapping representations, whereas the PC allows recalling a whole and specific memory from an incomplete input signal by complementing (activating) the missing components. The input signals projected from the sensory cortex and then, the entorhinal cortex processed it into a non-overlapping pattern in the dentate gyrus via the mossy fiber (PS). This non-overlapping pattern is then projected as distinct representations into the CA3 field of the hippocampus, after from which the representation could be retried from CA3 by diffusing activation to the cortex (PC, Rolls, 2016; Pishdadian et al., 2020). Modern approaches of neurocognition state that PS occurs beyond the hippocampus, all along the ventral stream (Kent et al., 2016; Cowell et al., 2019; Ekstrom and Yonelinas, 2020).

These modern approaches emphasize the content of information to be processed focusing on the visual modality for the moment. All visual representations are, for instance, processed hierarchically from the simplest processing within the visual cortex to the most complex processing in the hippocampus (Murray and Bussey, 1999; Saksida and Bussey, 2010). This representational-hierarchical model states that non-overlapping representations (PS) could be obtained by the conjunction of perceptual features along the ventral stream regions to the hippocampus (see Kent et al., 2016). Basic sensory interference between individual stimulus characteristics (e.g., lines and colors) can be resolved in the sensory cortex. When more perceptually complex features are introduced (e.g., objects), the interference is resolved at higher processing level (e.g., the perirhinal cortex -PrC). Finally, combinatorial codes (e.g., conjunctive representations of object scenes in a spatial context) are resolved in the hippocampus.

The representations are assumed to be widely distributed into a system where characteristics of objects and scenes are progressively combined as the processing progress hierarchically. Moreover, and consistently with the embodiment, memory representation would emerge from the activation of perceptual units, and thus, the distinction between memory and perception are no more relevant (see also, Graham et al., 2010). Thus, conjunctions would not only reduce memory interference, but would also eliminate the ambiguity of visually similar stimuli in supposedly non-memory tasks. Accordingly, the PrC is involved in processing of complex perceptual objects (Buckley et al., 2001; Bussey et al., 2002; Barense et al., 2007) and lesions to the PrC induce false recognition due to interference from similar memories (Burke et al., 2010; McTighe et al., 2010). Similarly, growing evidence indicates that the hippocampus underlies the processing of conjunctions of complex spatial information in memory (Girardeau et al., 2009) and in presumed non-mnemonic tasks (see, Lee et al., 2012).

Applied in aging, the functionally weakened sensory signals in the sensory cortex would increase perceptual/mnemonic interference as conjunctions would be less efficient along the ventral stream. Indeed, and on the contrary to the occipital cortex, the MTL is structurally affected by aging, both in the PrC (Ryan et al., 2012; Fidalgo et al., 2016) and the hippocampus (Fraser et al., 2015). As a result, conjunction processing is expected to be impoverished in older adults. This should increase interference mainly between similar memories (Surprenant et al., 2006; Ekstrom and Yonelinas, 2020). Actually, when the memories are sufficiently distinct (efficient PS or dissimilar memories), then the PC mechanism can easily complete a specific trace, but when the memory traces overlap, then the system will enter into an unstable attractor that may lead to the emergence of altered/confused information (inefficient PC) (Ekstrom and Yonelinas, 2020; Zotow et al., 2020). Furthermore, the contribution of conjunctive representations to reduce interference could be more important as the delay increases. Delay-dependent impairments after MTL damage result from deficient conjunction representations to resolve the ambiguity of simpler representations in lower-level regions that are more likely to be encountered during delay (Yonelinas, 2013). Therefore, the processing of similar information in aging could be impacted at low levels of similarity in long-term memory, while deficits would be evident at shorter time frames (e.g., short-term memory, perception) only at higher levels of similarity.

On a behavioral standpoint, less efficient PS in aging is mainly studied in memory using a *perceptual lure discrimination index*. In a study phase, the participant learns images. In a subsequent recognition task, these images (targets) are presented along with new images that are visually distinct (foils) or that are visually similar (lures) to the targets. The results show a linear decrease in perceptual lure discrimination as perceptual similarity increases in older adults (see Leal and Yassa, 2018). Older adults thus indicated more often having already seen a new image, especially when it is perceptually similar to a target (declining perceptual lure discrimination index). Similarly, false recognition was more likely for items in categories that are visually more similar than those that are more distinct (Boutet et al., 2019). It should be highlighted that the perceptual lure discrimination index is associated with more global cognitive functioning in older adults (Pishdadian et al., 2020). As such, the alteration of the conjunction processing in the MTL could have wider consequences than the specific memories.

## Distinctiveness of Memory Traces on Other Cognitive Domains

The consequence of reduced sensory processing in aging should not be limited to memory according to embodiment. According to the embodied and situated memory models Act-In (Activation-Integration, Versace et al., 2014), representations (e.g., semantic, episodic) emerge from the same sensorimotor components of the different memory traces. All experiences of the individual are supposed to be accumulated as memory traces. These traces are distributed across modal and heteromodal neuronal systems coding the multiple sensorimotor components of the experiences.

The different components of a given memory trace are bound together (Opitz, 2010), following the conjunction processing and therefore the PS. The binding allows the PC, described in Act-In as an intra-trace activation. A specific memory emerges when the activation does not propagate to similar traces (called inter-trace activation). Reversely, the activation of multiple similar traces (inter-trace activation) should produce categorial (non-specific/semantic) knowledge. A strong inter-trace diffusion achieves categorization by eliminating specific details and context of events (see Versace et al., 2009, 2014), mechanism underlined by the CA1 subregion of the hippocampus and neocortical upstream (Kumaran and McClelland, 2012). The intra-trace and inter-trace activations are mutually repulsive so strengthen or weaken one kind of activation should directly weaken or strengthen the other. Given that intra-trace activation is facilitated by distinctiveness, better simulations (higher resolution representations) should allow more activation of contextual details limiting the activation of common/similar components of other traces (inter-trace activation) (e.g., Ekstrom and Yonelinas, 2020). Furthermore, distinctiveness is also a function of the number of experiences accumulated, then more traces should increase the likelihood of their overlap. This, in turn, should increase the inter-trace activation. Consequently, the decline of perceptive and conjunctive processing associated with more traces (more events experienced by older adults) should induce less distinct traces in older adults (Vallet, 2015), then it should in return bias the dynamics of the simulation in favor of the inter-trace activation (less distinct processing).

This balance between specific and non-specific knowledge could be illustrated by the fact that older adults produce fewer internal (specific) details associated with more external (categorial) details when they recall an event compared to young adults (e.g., Levine et al., 2002). The effect of aging on the visual cortex and the hippocampus reduces specific details (neural dedifferentiation), while aging enhances categorial representations in the anterior temporal lobe (neural hyperdifferentiation) (Deng et al., 2021). Therefore, older adults exhibit relatively well-preserved performance in semantic tasks (see, Salthouse, 2010, for a review). However, a more qualitative analysis shows that they produce more categorial (e.g., cat) and less unique (e.g., botfly) semantic knowledge (Murphy and Castel, 2020). Their memory difficulties are then not limited to a given memory system, but rather occur due to the alteration of mechanisms involved in the emergence of specific knowledge. Thus, not only do older adults have less detailed specific knowledge in episodic tasks (specific knowledge, Greene and Naveh-Benjamin, 2020; Frankenberg et al., 2021), but they also less benefit from distinctiveness (see Smith, 2006) due to a more generic (categorial) and less distinct processing (Koutstaal and Schacter, 1997; Smith, 2006).

Aging might be characterized by an imbalance toward generic (non-specific) processing constrained by the weight of prior knowledge at the expense of specific processing. Compared to young adults, older people produced indeed less specific details and more generic details in a basic image description task and in future imagination task (Gaesser et al., 2011; Schacter et al., 2013). Yet, an integrated view of neurocognitive functioning suggests that deficits in modal (e.g., less specialization in occipital processing) and heteromodal (e.g., structural impairment of MTL) regions should alter the processing done within the connected regions as the prefrontal cortex (Davis et al., 2008). The neuromodulation deficit induces noise in the neural processing, including the prefrontal cortex, and leads to less specific and more general processing (Li et al., 2001). As the processes are less specific, and the similarity of the previous processes favors the automation of processes (see Logan, 1988), new processes are less likely to emerge. The same over-repeated processes will more likely emerge, making more rigid and less flexible other processing. This rigidity is found for their executive functions such as to change categorization rules in the Wisconsin tasks (e.g., Daigneault et al., 1992; Ashendorf and McCaffrey, 2008). Interestingly, the largest executive switching costs were found under conditions of ambiguous sensory stimuli and overlap between sets of responses (Mayr, 2001). This is consistent with the hypothesis that perceptual deficits reduce trace distinctiveness and lead to increased inter-trace activation, requiring more inhibition in prefrontal cortex (Li et al., 2001). Similar link between sensory functioning and inhibition is observed in young adults with degraded vision (e.g., cataract vision simulation, as found in pathological visual aging) in the Stroop task (see Monge and Madden, 2016 for a review). A more evidence comes from the Perceptual Lure Discrimination Index in the Mnemonic Similarity Task that is related to inhibition (Foster and Giovanello, 2020). Finally, it is noteworthy that the MocA (global measure of cognitive aging) were associated with lure discrimination performances in older adults (Pishdadian et al., 2020).

## Conclusion

Normal aging is characterized by sensory-perceptual (and motor) decline, on the one hand, and cognitive decline on the other. Further studies, using a longitudinal design, are required to fully explore the progressive sensory (motor) and cognitive changes occurring throughout life. Embodied cognition provides a theoretical framework explaining the possible these links given that any representation at the source of cognitive functioning remains grounded in these sensorimotor components. As such, memory and perception (and action) are functionally equivalent. This perspective investigated the neurophysiological mechanisms underlying these links. The sensory decline (organ level) should have a minimal impact, mainly on overlapping stimuli by impoverishment of the related signal. Higher perceptual decline should affect the simulation mechanism leading to the emergence of a less specific and detailed representation. Functional changes in the primary perceptual areas may reduce the benefit of distinct perceptual information, while structural changes in the MTL may reinforce of overlapping perceptual and memory information. Since embodied representation should be at the core of cognition, such changes should have wider cognitive consequences than memory. Thus, aging could be characterized by less specific and more rigid processing.

This perspective highlights the interest to study aging in an embodied cognition approach, which could represent an alternative to other theories of cognitive aging due to how the sensory (motor)- cognitive interactions are defined. The focus of the present article on perceptual-memory interactions also suggest that early sensory improvement and environmental enrichment could improve cognitive aging (Leon and Woo, 2018). Similar effects should also be found with motor and action interactions. We hope that this brief overview of the contribution of embodied cognition to characterize neurocognitive aging will encourage further investigation of cognitive functions in aging from an embodied perspective.

## Data Availability Statement

The original contributions presented in the study are included in the article/supplementary material, further inquiries can be directed to the corresponding author/s.

## Author Contributions

JM: conceptualization, writing – original draft, and project administration. GV: conceptualization, writing – review and editing, supervision, funding acquisition, and resources. SB: writing – review and editing. MI: writing – review and editing, and supervision. All authors contributed to the article and approved the submitted version.

## Conflict of Interest

The authors declare that the research was conducted in the absence of any commercial or financial relationships that could be construed as a potential conflict of interest.

## Publisher’s Note

All claims expressed in this article are solely those of the authors and do not necessarily represent those of their affiliated organizations, or those of the publisher, the editors and the reviewers. Any product that may be evaluated in this article, or claim that may be made by its manufacturer, is not guaranteed or endorsed by the publisher.
